# Accuracy of intraoperative frozen section in the diagnosis of ovarian neoplasms: Experience at a tertiary oncology center

**DOI:** 10.1186/1477-7819-4-12

**Published:** 2006-02-24

**Authors:** Amita Maheshwari, Sudeep Gupta, Shubhada Kane, Yogesh Kulkarni, Lt Col Bhupesh Kumar Goyal, Hemant B Tongaonkar

**Affiliations:** 1 Gynaecologic Oncology Service, Department of Surgical Oncology, Tata Memorial Hospital, Mumbai, India; 2 Department of Medical Oncology, Tata Memorial Hospital, Mumbai, India; 3 Department of Pathology, Tata Memorial Hospital, Mumbai, India; 4 Department of Obstetrics & Gynaecology, Armed Forces Research and Referral Hospital, New Delhi, India

## Abstract

**Background:**

Epithelial ovarian neoplasms are an important cause of morbidity and mortality in women. The surgical management of ovarian neoplasms depends on their correct categorization as benign, borderline or malignant. This study was undertaken to evaluate the accuracy of intra-operative frozen section in the diagnosis of various categories of ovarian neoplasms.

**Methods:**

Intraoperative frozen section diagnosis was retrospectively evaluated in 217 patients with suspected ovarian neoplasms who underwent surgery as primary line of therapy at our institution. This was compared with the final histopathologic diagnosis on paraffin sections.

**Results:**

In 7 patients (3.2%) no opinion on frozen section was possible. In the remaining 210 patients frozen section report had a sensitivity of 100%, 93.5% and 45.5% for benign, malignant and borderline tumors. The corresponding specificities were 93.2%, 98.3% and 98.5% respectively. The overall accuracy of frozen section diagnosis was 91.2%. The majority of cases of disagreement were in the mucinous and borderline tumors.

**Conclusion:**

Intraoperative frozen section has high accuracy in the diagnosis of suspected ovarian neoplasms. It is a valuable tool to guide the surgical management of these patients and should be routinely used in all major oncology centers.

## Background

Epithelial ovarian neoplasms are an important cause of morbidity and mortality in women. The three main categories of ovarian neoplasms are benign, borderline and malignant, which differ with respect to their biologic characteristics, management and prognosis. Tumors of 'borderline malignancy' are an important group characterized by some histologic features of malignancy (epithelial cell stratification, increased mitotic activity, nuclear atypia) but lack stromal invasion. They have an excellent long-term outcome even after conservative surgery. It is extremely helpful and sometimes critical to know intraoperatively the category of tumor one is dealing with, primarily to decide the extent of surgery. Benign and borderline tumors can be adequately treated with conservative surgery, which may involve preservation of fertility in younger women [1]. In contrast, malignant epithelial neoplasms usually require extensive surgery with total abdominal hysterectomy, bilateral salpingo-oophorectomy and omentectomy along with pelvic and retro-peritoneal lymphadenectomy and sampling from many peritoneal sites.

Preoperative imaging and tumor markers have only limited value in differentiating between these tumor categories [2,3]. Intraoperative frozen section diagnosis of ovarian tumors is widely used in making this distinction and to decide the surgical course. Therefore the accuracy of this technique is very important. It has been reported to have a good diagnostic accuracy in benign and malignant tumors, but a lower accuracy for borderline tumors [4]. The aim of the current study was to evaluate the accuracy of intra-operative frozen section in the diagnosis of ovarian neoplasms at our institution.

## Materials and methods

Case records of 241 patients with ovarian neoplasms who underwent frozen section diagnosis between 1997 and 2001 were retrospectively reviewed. During this time period, only patients with the following indications received intraoperative frozen section diagnosis:

1. Clinically benign looking tumors (on preoperative radiology or intraoperative inspection) with raised CA- 125.

2. Adnexal mass in a patient with a past history of malignancy at another site.

3. Young patients with ovarian neoplasms in whom fertility sparing surgery was planned.

The procedure for carrying out the frozen section diagnosis was as follows: A laparotomy was performed and the tumor was first removed. The unfixed tumor was immediately delivered to the frozen section laboratory situated in the operation theatre complex with all the clinical details of the patient. After gross examination of the tumor, sections were obtained from representative areas at the discretion of the pathologist. The number of sections frozen ranged from 1 to 4 and depended on the type and size of the tumor. In all tumors diagnosed as borderline at least 2 sections were frozen. This was done in the cryostat instrument. Seven to 8 μm sections were obtained and stained with hematoxylin-eosin and toluidine blue. All the sections were studied microscopically under low and high power by two pathologists. The frozen section diagnosis was conveyed to the surgical team who then proceeded with the appropriate surgery. The average time taken in the entire procedure (sending the sample to obtaining the result) was approximately 10 minutes. The frozen section diagnosis was categorized as one of the following: primary epithelial ovarian neoplasm – benign, borderline or malignant; primary ovarian germ cell tumor, metastatic carcinoma to ovary, benign non-neoplastic conditions and no definite opinion possible. Frozen section diagnosis was compared to the final paraffin section diagnosis.

### Statistical analysis

The overall accuracy was defined as the total number of agreements between the frozen section and the final diagnosis divided by the total number of tests performed. For the purposes of this study the final histopathologic diagnoses were assumed to be correct. The sensitivity and specificity and predictive values of frozen section for the diagnosis of various categories of neoplasms were calculated using the standard 2 × 2 method shown in Table 1.

**Table 1:** 

	**Control**		
	
**Test**	Positive	Negative	Total
Positive	a	b	a + b
Negative	c	d	c + d
Total	a + c	b + d	N

Sensitivity: a/(a + c)

Specificity: d/(b + d)

Positive predictive value (PPV): a/(a + b)

Negative predictive value (NPV): d/(c + d)

## Results

Two hundred and forty one patients of ovarian neoplasms underwent frozen section diagnosis. The median age of the patients was 44 years (10–80 years). Twenty four patients had their frozen sections performed on tissues other than ovarian and were excluded from further analysis. Of the remaining 217 patients, the frozen section diagnosis agreed with the final histopathology as to the classification of benign, borderline or malignant in 198 cases (91.2%) and disagreed in 12 cases (5.5%). In 7 cases (3.2%) no definite opinion was possible on frozen section. The reasons for deferment of frozen section diagnoses were as follows: 1) Infarcted and necrotic lesions, and 2) Cystic lesions devoid of lining epithelium.

Seven deferred cases were excluded from the further analysis which was performed on the remaining 210 cases. Table 2 is a 3 × 3 table showing the results in 210 patients in whom a frozen section diagnosis was given. It should be noted that the diagnosis 'benign' included benign ovarian neoplasms and benign non-neoplastic conditions like corpus luteal cysts, endometriosis etc. The overall accuracy of the test was 94.28% (198 of 210 cases).

**Table 2:** The results of frozen and paraffin sections in various categories of ovarian neoplasms (n = 210)

**Frozen Diagnosis**	**Final Diagnosis (Paraffin)**			
	**Benign**	**Borderline**	**Malignant**	**Total**

**Benign**	107	04	03	114
**Borderline**	0	05	03	08
**Malignant**	0	02	86	88
**Total**	**107**	**11**	**92**	**210**

Table 3 shows the performance of frozen section in the 3 categories of ovarian tumors in our patients. Frozen section had a high sensitivity, specificity, positive and negative predictive values for benign conditions (including benign tumors) and malignant ovarian tumors, (Table 3). However, frozen section had low sensitivity (45.5%) and positive predictive value (62.5%) for borderline tumors while retaining high specificity and negative predictive value in them.

**Table 3:** Sensitivity, Specificity, PPV & NPV of Frozen Section

	**Benign**	**Malignant**	**Borderline**
**Sensitivity**	100%	93.4%	45.4%
**Specificity**	93.2%	98.3%	98.3%
PPV*	93.8%	97.7%	62.5%
NPV**	100%	95.1%	97%

* Positive Predictive Value ** Negative Predictive Value

## Discussion

Correct intraoperative histologic assessment of an ovarian mass is crucial to select an appropriate surgical procedure and avoid under- and over-treatment of the patient. The results of the present study show that frozen section analysis has a high overall accuracy for the diagnosis of ovarian neoplasms (91.2%). Most studies have reported the accuracy of frozen section from 90% to 97% [3,5-8]. This figure would obviously depend on the expertise of the pathologists but the overall inaccuracy should be consistently less than 10% for this procedure to be useful in large tertiary centers dealing with ovarian neoplasms. Furthermore, frozen section diagnosis was possible in 210 of the 217 patients (96.8%) in whom this procedure was done. Evidently this procedure is technically feasible in the overwhelming majority of patients.

Further analysis shows that the test fared very well on all 4 conventional indices (sensitivity, specificity and predictive values) in benign conditions and malignant tumors. It is evident that frozen section does not miss the diagnosis of benign and malignant ovarian tumors in the vast majority of patients (high sensitivity) and furthermore frozen section diagnosis of benign and malignant ovarian tumors are correct in the vast majority (high predictive values). Thus only in 3 patients out of the 114 called benign by frozen section, the diagnosis was changed to malignant tumor on the final histopathology (with the implication that these patients might have been under-treated at initial surgery). All these were low grade, mucinous tumors on final histology. In only 2 patients out of the 88 patients diagnosed as malignant on frozen section, the final diagnosis was changed to borderline while none was changed to benign (with the implication that these patients might have been over treated at initial surgery). A recently published meta-analysis of eighteen studies comparing frozen section diagnosis of ovarian pathology with the final histopathology showed the sensitivity of frozen section for benign and malignant lesions to vary from 65% to 97% and 71% to 100% respectively [9]. The same analysis showed the specificity to vary from 97% to 100% and 98.3% to 100% for benign and malignant lesions respectively.

However the situation is different in borderline tumors. The sensitivity and the positive predictive valve of frozen section were 45.5% and 62.5% respectively in the current study. The reported sensitivity of frozen section in borderline tumors varies from 0–87% in various studies [5-8,10-12]. Various reasons have been advanced for the relative inaccuracy of frozen section in the diagnosis borderline tumors. In a large borderline tumor there may be only a few foci of frank malignancy that may require large number of frozen sections samples for diagnosis. This is very labor intensive and usually beyond the capabilities of most laboratories. It has also been suggested that it may be more difficult to diagnose borderline mucinous tumors compared to borderline serous tumors because of their larger average size [5,12]. In our cases, out of the 11 patients with a final diagnosis of borderline tumors, 5 were correctly identified by frozen section. Four were incorrectly labeled as benign, which may not have critical therapeutic implications since patients with borderline tumors also do well with conservative surgery [1]. Of greater therapeutic implication was the incorrect frozen report of malignancy in 2 of these 11 patients since extensive surgery is not required in borderline tumors, particularly in early stages.

A recently published systematic review of 14 studies on the accuracy of frozen section in the diagnosis of ovarian tumors also concluded that frozen section has high accuracy rates for the diagnosis of benign and malignant ovarian tumors but the accuracy rates in borderline tumors remains relatively low [4].

## Conclusion

We conclude that frozen section is an accurate and useful test in the intraoperative evaluation of patients with suspected ovarian neoplasms. Its results can be used to guide the type and extent of surgery, especially in institutions with experienced pathologists. The accuracy of frozen section is very high for benign and malignant tumors but lower for borderline tumors. Every effort should be made to establish this procedure in institutions that treat large numbers of patients with ovarian tumors.

## Competing interests

The author(s) declare that they have no competing interests.

## Authors' contributions

Dr. Maheshwari conceived the study and was the involved in its planning, data collection analysis and writing. Dr. Gupta was involved with the statistical analysis and writing the study. Drs. Kane, Kulkarni, Goyal and Tongaonkar critically reviewed the paper and were involved in the preparation of the final manuscript.
